# Evaluating COVID-19 severity prediction and immune dynamics with NULISAseq: Insights from the IMPACC study

**DOI:** 10.1093/jimmun/vkaf263

**Published:** 2025-10-30

**Authors:** Koji Abe, Tyson H Holmes, Tran T Nguyen, IMPACC Network, Koji Abe, Tyson H Holmes, Tran T Nguyen, Seunghee Kim-Schulze, Ofer Levy, Lindsey R Baden, Esther Melamed, Lauren I R Ehrlich, Grace A McComsey, Rafick P Sekaly, Charles B Cairns, Elias K Haddad, Albert C Shaw, David A Hafler, Ruth R Montgomery, David B Corry, Farrah Kheradmand, Mark A Atkinson, Scott C Brakenridge, Nelson I Agudelo Higuita, Jordan P Metcalf, Catherine L Hough, William B Messer, Bali Pulendran, Kari C Nadeau, Mark M Davis, Ana Fernandez-Sesma, Viviana Simon, Monica Kraft, Chris Bime, Carolyn S Calfee, David J Erle, Joanna Schaenman, Elaine F Reed, Al Ozonoff, Bjoern Peters, Steven H Kleinstein, Alison D Augustine, Joann Diray-Arce, Patrice M Becker, Nadine Rouphael, Holden T Maecker, Seunghee Kim-Schulze, Ofer Levy, Lindsey R Baden, Esther Melamed, Lauren I R Ehrlich, Grace A McComsey, Rafick P Sekaly, Charles B Cairns, Elias K Haddad, Albert C Shaw, David A Hafler, Ruth R Montgomery, David B Corry, Farrah Kheradmand, Mark A Atkinson, Scott C Brakenridge, Nelson I Agudelo Higuita, Jordan P Metcalf, Catherine L Hough, William B Messer, Bali Pulendran, Kari C Nadeau, Mark M Davis, Ana Fernandez-Sesma, Viviana Simon, Monica Kraft, Chris Bime, Carolyn S Calfee, David J Erle, Joanna Schaenman, Elaine F Reed, Al Ozonoff, Bjoern Peters, Steven H Kleinstein, Alison D Augustine, Joann Diray-Arce, Patrice M Becker, Nadine Rouphael, Holden T Maecker

**Affiliations:** Stanford University School of Medicine, Palo Alto, CA, United States; Stanford University School of Medicine, Palo Alto, CA, United States; Stanford University School of Medicine, Palo Alto, CA, United States; Icahn School of Medicine at Mount Sinai, New York, NY, United States; Precision Vaccines Program, Boston Children’s Hospital, Harvard Medical School, Boston, MA, United States; Brigham and Women’s Hospital, Harvard Medical School, Boston, MA, United States; The University of Texas at Austin, Austin, TX, United States; The University of Texas at Austin, Austin, TX, United States; Case Western Reserve University and University Hospitals of Cleveland, Cleveland, OH, United States; Case Western Reserve University and University Hospitals of Cleveland, Cleveland, OH, United States; Tower Health Hospital, Drexel University, Philadelphia, PA, United States; Tower Health Hospital, Drexel University, Philadelphia, PA, United States; Yale School of Medicine, New Haven, CT, United States; Yale School of Medicine, New Haven, CT, United States; Yale School of Medicine, New Haven, CT, United States; Baylor College of Medicine and the Center for Translational Research on Inflammatory Diseases, Houston, TX, United States; Baylor College of Medicine and the Center for Translational Research on Inflammatory Diseases, Houston, TX, United States; University of Florida, Gainesville, FL, United States; University of Florida, Gainesville, FL, United States; Oklahoma University Health Sciences Center, Oklahoma City, OK, United States; Oklahoma University Health Sciences Center, Oklahoma City, OK, United States; Oregon Health & Science University, Portland, OR, United States; Oregon Health & Science University, Portland, OR, United States; Stanford University School of Medicine, Palo Alto, CA, United States; Stanford University School of Medicine, Palo Alto, CA, United States; Stanford University School of Medicine, Palo Alto, CA, United States; Icahn School of Medicine at Mount Sinai, New York, NY, United States; Icahn School of Medicine at Mount Sinai, New York, NY, United States; University of Arizona, Tucson, AZ, United States; University of Arizona, Tucson, AZ, United States; University of California, San Francisco, San Francisco, CA, United States; University of California, San Francisco, San Francisco, CA, United States; David Geffen School of Medicine, University of California, Los Angeles, Los Angeles, CA, United States; David Geffen School of Medicine, University of California, Los Angeles, Los Angeles, CA, United States; Clinical and Data Coordinating Center, Precision Vaccines Program, Boston Children’s Hospital, Harvard Medical School, Boston, MA, United States; La Jolla Institute for Immunology, La Jolla, CA, United States; Yale School of Medicine, New Haven, CT, United States; National Institute of Allergy and Infectious Diseases, National Institutes of Health, Bethesda, MD, United States; Clinical and Data Coordinating Center, Precision Vaccines Program, Boston Children’s Hospital, Harvard Medical School, Boston, MA, United States; National Institute of Allergy and Infectious Diseases, National Institutes of Health, Bethesda, MD, United States; Emory School of Medicine, Atlanta, GA, United States; Stanford University School of Medicine, Palo Alto, CA, United States

**Keywords:** cytokines, human, inflammation, molecular biology, viral

## Abstract

The National Institutes of Health–funded IMPACC (IMmunoPhenotyping Assessment in a COVID-19 Cohort) evaluated longitudinal clinical and immunological features of human patients hospitalized for COVID-19. This study focuses on comparing the novel NULISAseq assay with the Olink platform using a subset of participants to assess their efficacy in predicting COVID-19 severity and understanding immune response dynamics. Our findings reveal that NULISAseq could provide superior detectability and dynamic range across various targets. Elastic net analysis demonstrated that specific proteins, including amphiregulin, effectively predict COVID-19 severity from sera at admission (samples drawn within 96 h of admission), with a test area under the curve of 0.84. Longitudinal analysis identified significant differences in multiple targets, including IL-5 and interferons, between low- and high-severity groups over time. Additionally, association rule mining suggested potential early markers predictive of later immune cell changes. These findings emphasize the potential of NULISAseq for comprehensive profiling, early prediction, and identification of targeted therapeutic interventions in COVID-19.

## Introduction

The National Institutes of Health (NIH)–funded IMPACC (IMmunoPhenotyping Assessment in a COVID-19 Cohort) evaluated longitudinal clinical and immunological features of 1,164 patients hospitalized for COVID-19. This comprehensive study employed multiple highly multiplexed assays for genomic, protein, cellular, and viral phenotyping.^1^ Previous studies from IMPACC identified cellular and molecular signatures, including serum inflammatory protein markers measured by a proximity extension assay (Olink), associated with severe COVID-19 outcomes.^2–5^

NULISAseq is a novel proximity ligation assay utilizing capture and release steps to minimize background noise caused by nonspecific binding, thereby achieving high detectability.^6^ In our preliminary evaluation of three distinct immunoassay platforms—Luminex, Olink, and NULISAseq—using a small subset of IMPACC samples (23 patients), NULISAseq demonstrated superior detectability.^7^

The objective of this study was to investigate the performance of NULISAseq with a larger subset of the IMPACC dataset comprising 503 participants. This included a longitudinal analysis and prediction of COVID-19 severity at admission (i.e. with samples drawn within 96 h of admission). Additionally, we utilized association rule mining (ARM) to provide insights into the relationships between cytokine levels from NULISAseq and cell subset distributions obtained by CyTOF, revealing patterns that could inform our understanding of disease mechanisms and potentially highlight targets for therapeutic intervention. We also compared the results with those obtained using the Olink platform to provide a comprehensive assessment of NULISAseq’s capabilities.

## Methods

### Ethics

National Institute of Allergy and Infectious Diseases staff conferred with the Department of Health and Human Services Office for Human Research Protections regarding potential applicability of the public health surveillance exception [45CFR46.102(l)(2)] to the IMPACC study protocol. The Office for Human Research Protections concurred that the study satisfied criteria for the public health surveillance exception, and the IMPACC study team sent the study protocol, and participant information sheet for review, and assessment to Institutional Review Boards (IRBs) at participating institutions. Twelve institutions elected to conduct the study as public health surveillance, while 3 sites with prior IRB-approved biobanking protocols elected to integrate and conduct IMPACC under their institutional protocols (University of Texas at Austin, IRB 2020-04-0117; University of California San Francisco, IRB 20-30497; University Hospitals Cleveland Medical Center at Case Western Reserve University, IRB STUDY20200573) with informed consent requirements. Participants enrolled under the public health surveillance exclusion were provided information sheets describing the study, samples to be collected, and plans for data de-identification, and use. Those that requested not to participate after reviewing the information sheet were not enrolled. In addition, participants did not receive compensation for study participation while inpatient and subsequently were offered compensation during outpatient follow-ups.

### Cohort

The IMPACC cohort is described in detail elsewhere.^2^^,^^7^^,^^8^ Briefly, the IMPACC study aimed to investigate the clinical and immunological characteristics of COVID-19 in hospitalized patients. The study enrolled participants from 20 hospitals affiliated with 15 institutions across the United States between May 2020 and March 2021. All patients included in the study were unvaccinated, providing a unique perspective on the immune response and disease progression in the absence of vaccine-induced immunity. Serum samples were collected at multiple time points during hospitalization, including at enrollment and subsequently on days 4, 7, 14, 21, and 28, as well as at 3, 6, 9, and 12 mo posthospitalization. These visits were designated as visits 1 to 10. Additional samples were collected within 24 and 96 h (escalation visits 1 and 2) when a participant required escalation of care or was readmitted to the hospital prior to visit 6. Escalation visits were excluded from statistical analyses (longitudinal analysis and prediction at admission) because escalation visits were not planned visits, but rather were based on clinical status. In contrast, the use of purely planned visits provided us with a more unbiased view of the course of disease. The disease progression was categorized into 5 distinct trajectories to capture the variability in clinical outcomes comprehensively.^8^ These trajectories were defined as follows: trajectory 1 indicated a brief hospital stay with a median duration of 3 d; trajectory 2 corresponded to an intermediate hospital stay with a median duration of 7 d; trajectory 3 represented an intermediate hospital stay, also with a median duration of 7 d, but included discharge limitations; trajectory 4 involved prolonged hospitalization with a median duration of 20 d; and trajectory 5 was associated with a fatal outcome.^8^

The subset from the IMPACC study utilized in the analysis with NULISAseq comprised 503 COVID-19 patients, with serum samples collected at up to 12 distinct time points (10 scheduled visits and 2 escalation visits), resulting in a total of 1,851 samples (mean age 57.08 yr, 200 females) (detailed in Table 1). In the ARM analysis, the number of shared participants was 469 for the combination of NULISAseq with CyTOF data, and 1,021 for the combination of Olink with CyTOF data, respectively.

**Table 1.** COVID-19 sample demographics (N = 503).

Age, yr	57.08 ± 14.64
Female)	200 (39.8)
Trajectory group	
1 (least severe)	66
2	96
3	145
4	145
5 (most severe)	51
Visits (N = 1,851)	
Enrollment (visit 1)	480
Day 4 (visit 2)	351
Day 7 (visit 3)	246
Day 14 (visit 4)	158
Day 21 (visit 5)	79
Day 28 (visit 6)	134
3 mo (visit 7)	67
6 mo (visit 8)	94
9 mo (visit 9)	114
12 mo (visit 10)	105
Escalation visit 1	12
Escalation visit 2	11

Values are mean ± SD, n (%), or n.

### Sample preparation

According to the IMPACC protocol, serum samples were processed and frozen within 6 h of collection. All specimens were stored at −80°C until they were thawed for use in each assay. Specifically, samples used in the Olink assay were subjected to one freeze-thaw cycle, whereas those in the NULISAseq assay underwent three freeze-thaw cycles. The samples were employed without any filtration or modification. This study received approval from the Stanford University School of Medicine IRB.

### Immunoassay platforms

Alamar’s NULISAseq is described in detail elsewhere.^6^^,^^7^ In brief, the NULISAseq inflammation panel primarily targets inflammation and immune response-related cytokines and chemokines. Total number of available protein targets in NULISAseq was 247 (current commercial assay has 250 targets). The capture antibody is conjugated with partially double-stranded DNA that includes a poly(A) tail and a target-specific barcode, while the detection antibody is attached to another partially double-stranded DNA containing a biotin group and a corresponding target-specific barcode. When both antibodies are incubated with a sample containing the target molecule, an immunocomplex is formed. These immunocomplexes are captured and washed by dT-poly(A) hybridization and salt concentration. A set of paramagnetic streptavidin-coated beads is then introduced to capture the immunocomplexes again, ensuring the removal of free unbound capture antibodies and yielding essentially pure immunocomplexes on the beads. Next, a ligation mix is added to allow the ligation of the proximal ends of DNA attached to the paired antibodies, generating a new DNA reporter molecule with unique target-specific barcodes. The levels of these DNA reporters are quantified through next-generation sequencing. Data normalization was performed using an internal control spiked into each sample well. The data were then rescaled and log2 transformed to produce NULISA protein quantification (NPQ) units, which were used for subsequent statistical analyses.

For the Olink immunoassay, samples were analyzed using the Olink Target 96 Inflammation panel, which detects 92 proteins linked to inflammatory conditions, following the manufacturer’s instructions. Briefly, an incubation mix containing pairs of oligonucleotide-labeled antibodies specific to each protein is added to the samples and incubated for 16 h at 4 °C. Each protein is targeted by 2 distinct epitope-specific antibodies to enhance assay specificity. The presence of the target protein allows the formation of a double-stranded oligonucleotide polymerase chain reaction (PCR) target. The next day, an extension mix initiates the generation of amplicons through PCR in 96-well plates. To detect specific proteins, a Dynamic Array integrated fluidic circuit 96 × 96 chip is primed and loaded with 92 protein-specific primers, sample amplicons, interplate controls, and negative controls. Real-time quantitative PCR is performed using a Biomark system (Fluidigm). Data are processed with real-time PCR analysis software employing the ΔΔCt method and Normalized Protein eXpression (NPX) manager, where a unit of NPX difference indicates a doubling of the protein concentration.

### Platform correlation analysis for technical insights

To investigate the performance of NULISAseq compared with the Olink platform, we utilized a subset of the IMPACC cohort data from visit 1. Both the NULISAseq and Olink datasets were merged and filtered to identify shared participants (n = 438). To ensure comparability, we verified that the protein target names in both datasets were consistent with a predefined target key based on UniProt ID. Rows with missing values were removed from both datasets. For the correlation analysis, proteins common to both datasets were identified, and scatter plots were generated to visualize the relationships between NULISAseq and Olink measurements. The correlation coefficients were calculated using the Spearman method. The analysis was performed using R packages reshape2,^9^ dplyr,^10^ and lattice.^11^

### Prediction at admission and longitudinal analysis for clinical insights

For longitudinal analysis, separately for each protein, NPQ (NULISAseq) or NPX (Olink) readouts were regressed on a natural cubic spline for days elapsed since admission, trajectory group (1 to 3 vs. 4 and 5), their interaction, a natural cubic spline for age, and enrollment site using linear quantile mixed models^12^ for modeling trends in the median, with a random intercept per participant for the longitudinal structure. The interaction term permitted the estimated spline effects to differ between trajectory groups, allowing for a wide range of differences in temporal trends in medians between groups. A likelihood ratio test was performed for the interaction term to assess if trends across time differed between trajectory groups. For prediction at admission, the data were then randomly partitioned 60% for training and 40% for testing, with random partitioning stratified on enrollment site. In the training data, trajectory group (1 to 3 vs. 4 and 5) was multivariately regressed on NPQ or NPX readouts for all proteins using the elastic net.^13^ Cross-validation was randomly repeated 30 times and the average penalty term employed in the final fit. In the test data, trajectory group (1 to 3 vs. 4 and 5) was multivariately regressed on NPQ or NPX readouts using a generalized linear model^14^ for those proteins selected by the elastic net in the training data. Also in the test data, an area under the curve estimate was obtained along with the corresponding receiver operating characteristic curve. Statistical analyses were conducted in R^15^ using packages reshape2,^9^ lqmm,^12^^,^^16^ mutoss,^17^ lattice,^11^ glmnet,^18^^,^^19^ and pROC.^20^ The *P* values from the linear quantile mixed models were adjusted^21^^,^^22^ to control the false discovery rate at 5%. Eighty-one NPQ values were missing from some proteins on one plate, which was excluded from all statistical analyses.

### Association rule mining for biological insights

We integrated NULISAseq visit 1 data with CyTOF data from visits 1 and 6 to investigate the temporal dynamics of the immune response within the first month of admission. The number of samples in each combination of visits from NULISAseq and CyTOF data is shown in Table S1. To prepare the datasets for ARM, we employed a clustering-based method known as 1-dimensional k-means (k = 4) to discretize continuous variables into groups: Q1, Q2, Q3, and Q4. Following this discretization, a subset of data columns was filtered to ensure the presence of all required cluster categories, thereby identifying and excluding columns missing any categories to retain only complete cases for analysis. The filtered data was then split based on severity categories (trajectory groups 1 to 3 and 4 and 5) to facilitate a more detailed analysis of the different severity groups. A log2 transformation was applied to the cell subset columns across datasets to ensure that the data was appropriately scaled for subsequent analysis.

To conduct ARM, we prepared the datasets using a bootstrapping approach to enhance reproducibility and reliability, as reported previously.^23^ For each dataset and severity category, we created bootstrap samples by repeatedly sampling with replacement from the original dataset. Specifically, for each seed value, we generated multiple bootstrap samples to ensure diverse and representative sampling of the data. The number of bootstraps per seed was 100, and 10 different seeds were utilized. Each bootstrap sample was then transformed into transaction data suitable for ARM, which was performed to discover associations between cytokine levels and cell subset distributions. We defined specific combinations of interest, such as associations between high cytokine levels (Q4) and low cell subset levels (Q1), and vice versa. The support threshold was 0.3 and confidence level was 0.8 to focus on the most significant and reliable rules. The ARM algorithm was run on each bootstrap sample to extract rules that meet the specified criteria. The rules across all bootstrap samples were collated to identify common rules that were consistently supported. This approach allowed us to identify association rules that were not only meaningful but also reproducible and robust across different subsamples of the data. To refine the results of the association rule mining, we filtered the identified rules based on their lift values, considering only those with a lift >2.0. Table S2 shows the number of rules found in each case: case A (NULISAseq: visit 1; CyTOF: visit 1) and case B (NULISAseq: visit 1; CyTOF: visit 6). We also integrated Olink visit 1 data with CyTOF data from visits 1 and 6 to compare the results. Table S3 shows the number of samples in each combination of visits from Olink and CyTOF data.

The analyses were conducted using R packages dplyr,^22^ purrr,^24^ and arules.^25^

## Results

### Technical insights

#### Correlation with Olink for 65 common targets

**Figure 1.**
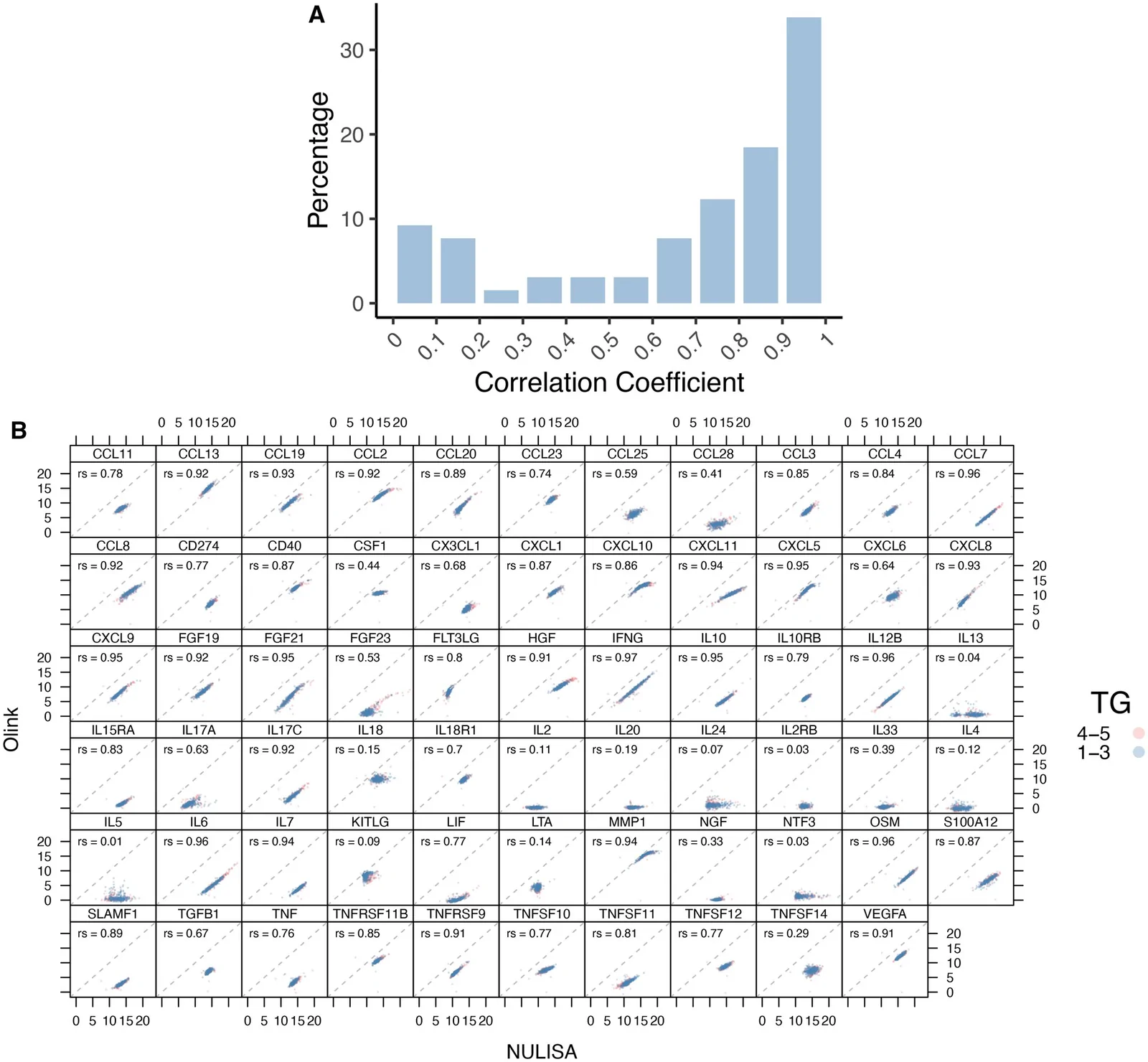
Correlation of measured values across platforms for 65 common targets using samples of COVID-19 positive participants at visit 1 (n = 438). (A) Distribution of Spearman’s rank correlation coefficient for all common targets. (B) Correlation and Spearman’s coefficient for each target. Trajectory groups (TGs) are highlighted as blue (1–3) and red (4 and 5).

A majority of the common targets showed strong positive correlations (rs = 0.7 to 1.0), while some targets indicated moderate (rs = 0.5 to 0.7) or weak (rs = 0.0 to 0.5) correlations (Fig. 1A, B). Moderate correlation targets include CCL25, CX3CL1, CXCL6, FGF23, IL-17A, IL-18R1, and TGFβ-1, while weak correlation targets were CCL28, CSF1, IL-13, IL-18, IL-2, IL-20, IL-24, IL-2RB, IL-33, IL-4, IL-5, KITLG, LTA, NGF, NTF3, and TNFSF14. Some targets with moderate and weak correlations (e.g. CXCL6, IL-18R1, IL-18, IL-2RB) exhibited less variability in values across both platforms. Conversely, other targets with weak correlations (e.g. IL-13, IL-2, IL-24, NGF, NTF3) displayed low signals when measured by Olink but demonstrated a range of signals when assessed using NULISAseq. Additionally, some targets with strong correlations (e.g. CCL2, CXCL10, MMP1) demonstrated less variability in Olink measurements for observations in which NULISAseq detected notable changes in signal. These observations suggest that NULISAseq possesses robust dynamic range and/or detectability for these specific targets. Figure 1B also illustrates that certain targets, such as CCL7 and IL-6, exhibited relatively higher levels in trajectory groups 4 and 5 across a subset of samples on both the Olink and NULISAseq platforms. This pattern suggests that these targets could serve as signature proteins indicative of disease severity, a hypothesis we explored further in both longitudinal and analyses at admission.

### Clinical insights

#### Prediction at admission

**Figure 2.**
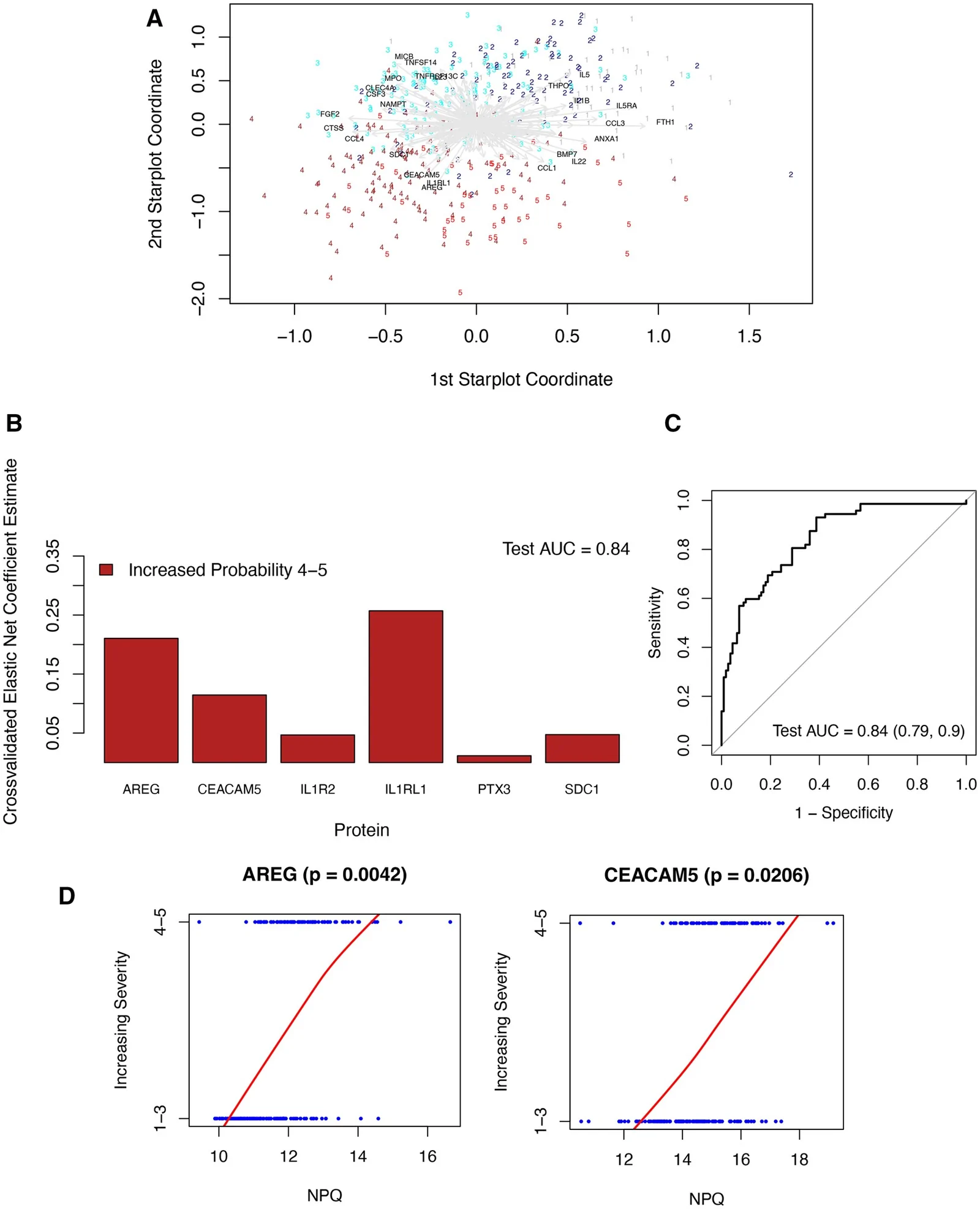
Prediction at admission using NULISAseq data. (A) Penalized supervised star plot showing separation of trajectory groups in 2D space. Vectors are the projection coefficients for the most prominent targets. Larger coefficients lead more to separation of trajectory groups. (B) Factors that separate trajectory groups estimated by elastic net. (C) Area under the receiver-operating characteristic curve (AUC) in the test data estimated using proteins from (B). A 95% confidence interval is provided for the AUC estimate. (D) Factors that separate trajectory groups at admission by generalized linear model. Red line is a fit of a lowess smooth curve. n = 183 (trajectory groups 1–3: n = 111; trajectory groups 4 and 5: n = 72).

We utilized penalized supervised star plot^26^ and elastic net methodologies for the prediction of trajectory groups at admission. The star plot visibly delineated the trajectory groups in ascending order of severity, highlighting several proteins that contribute to this separation (Fig. 2A). Figure 2B and C illustrates that six target proteins (AREG, CEACAM5, IL-1R2, IL-1RL1, PTX3, and SDC1) at admission effectively predict severity with a test area under the curve estimate of 0.84. This demonstrates that elevations in all six proteins at admission are correlated with increased severity. Notably, among the proteins selected by elastic net, AREG and CEACAM5 significantly predict severity in the test dataset (*P* < 0.021), as shown in Fig. 2D.

**Figure 3.**
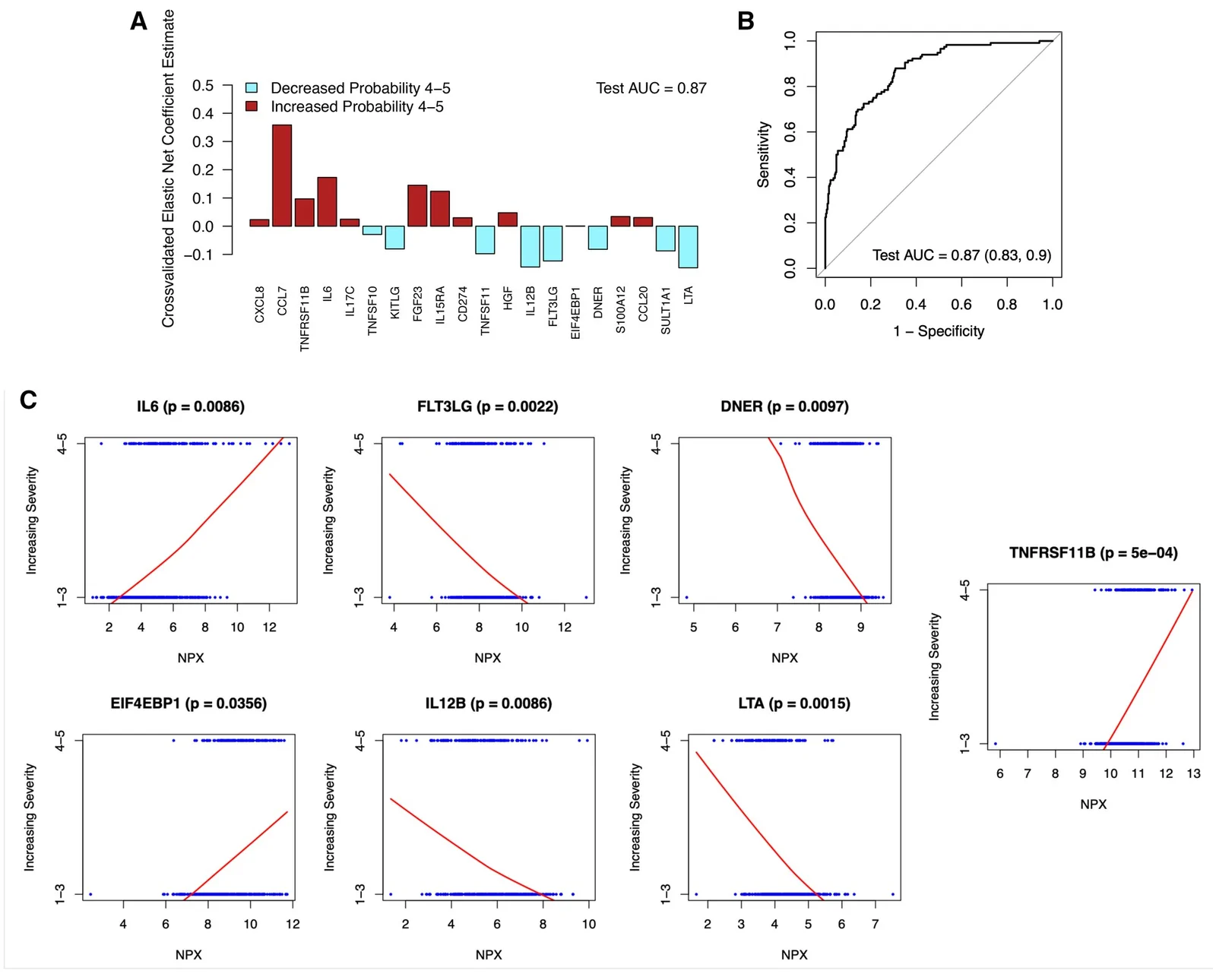
Prediction at admission by elastic net using Olink data. (A) Factors that separate trajectory groups. (B) Area under the receiver-operating characteristic curve (AUC) in the test data. A 95% confidence interval is provided for the AUC estimate. (C) Factors that separate trajectory groups at admission by generalized linear model. Red line is a fit of a lowess smooth curve. n = 424 (trajectory groups 1–3: n = 308; trajectory groups 4 and 5: n = 116).

We also applied the elastic net for prediction at admission using Olink data. Twenty proteins at admission jointly predict disease severity with an estimated test AUC of 0.87 (Fig. 3A, B). The direction of the effects among these proteins varies, with some increasing and others decreasing the probability of severe disease. Among the proteins selected by elastic net during training, TNFRSF11B, IL-6, IL-12B, FLT3LG, EIF4EBP1, DNER, and LTA emerged as significant predictors (*P* < 0.036) of the probability of severe disease in the test data (Fig. 3C).

### Longitudinal analysis

**Figure 4.**
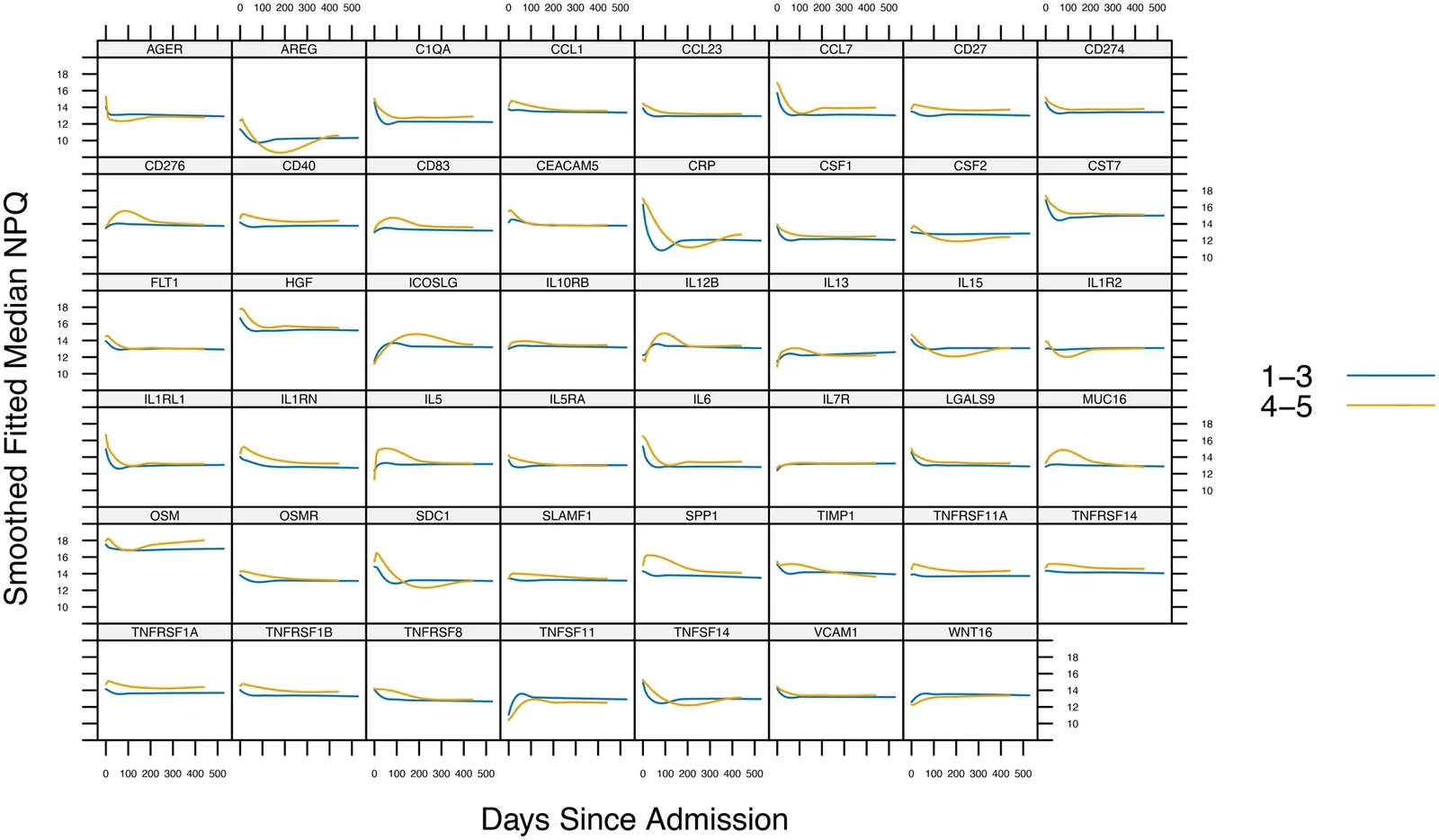
Longitudinal analysis with linear quantile mixed models for modeling trends in the median using NULISAseq data. Statistically significant proteins with likelihood ratio test statistics in the upper quartile are shown. Due to high statistical power, even subtle differences could be detected as significant (e.g. IL7R). The smooth fitted median NPQ represents the loess-smoothed LQMM fitted values of median NULISA expression, with smoothing over age and enrollment site.

A total of 190 proteins exhibited statistically significant differences in longitudinal trends between severity groups (Fig. S1). Although these proteins showed distinct patterns, the fitted median NPQ values tended to be higher for trajectory groups 4 and 5 for the majority of these proteins. This indicates an upregulation of inflammatory cytokines in the more severe groups, suggesting a correlation between elevated levels of these cytokines and increased disease severity. Based on the visual separation between the trajectory groups, the most pronounced differences were seen in AREG, CD276, ICOSLG, IL-12B, CRP, IL-5, IL-6, MUC16, and SPP1 (Fig. 4). Notably, many of these prominent proteins exhibited elevated values at the beginning of the observation period, followed by a resolution to lower levels at later time points. Figure S2 further demonstrates that these longitudinal differences begin to emerge near the time of admission, within the first 30 d, as observed for proteins such as IL-10 and IL-5. Interestingly, IFNα-1/13 and IFNα-2, which were reported as markers for COVID-19 severity,^27^ exhibited higher levels at earliest time points for trajectory groups 1 to 3.

Using Olink data, 79 proteins exhibited statistically significant differences in longitudinal trends between severity groups (Fig. S3A). Among the proteins with the most pronounced differences were CDCP1, FGF23, and IL-6. When focusing on the first 30 d after admission, the largest differences in longitudinal trends between severity groups were observed for proteins such as CCL7 and IL-6 (Fig. S3B).

### Biological insights

#### Association between cytokines by NULISAseq and cell subsets by CyTOF

ARM was employed to investigate the temporal dynamics of the immune response in COVID-19 patients within the first month of admission. Data from different visits were combined, discretized into groups using a clustering-based method, and then analyzed for associations between cytokines and cell subsets. For the severe group (trajectory groups 4 and 5) with a lift threshold of 2.0, several robust association rules were identified (Table 2). Interestingly, no rules met the same criteria for the mild group (trajectory groups 1 to 3), underscoring the distinct immune profiles between the severity categories.

**Table 2.** Rules found by ARM for trajectory groups 4 and 5 from NULISAseq (visit 1) and CyTOF (visit 6).

Association rule	Plain language translation
{CD4..T.Cell..EMRA.CD57hi.=Q1} => {CXCL1=Q1}	Low levels of CD4+ EMRA CD57hi T cells are associated with low levels of CXCL1 cytokine
{CD4..T.Cell..EMRA.CD57hi.=Q1} => {IL16=Q1}	Low levels of CD4+ EMRA CD57hi T cells are associated with low levels of IL16 cytokine
{CD4..T.Cell..EMRA.CD57hi.=Q1} => {TAFA5=Q1}	Low levels of CD4+ EMRA CD57hi T cells are associated with low levels of TAFA5 protein
{CD4..T.Cell..EMRA.CD57hi.=Q1} => {TIMP2=Q1}	Low levels of CD4+ EMRA CD57hi T cells are associated with low levels of TIMP2 protein
{CD4..T.Cell..EMRA.CD57hi.=Q1} => {TNFSF8=Q1}	Low levels of CD4+ EMRA CD57hi T cells are associated with low levels of TNFSF8 cytokine
{CD8..T.Cell..CD161..MAIT.=Q1} => {SPP1=Q4}	Low levels of CD8+ CD161+ MAIT cells are associated with high levels of SPP1 protein
{CD8..T.Cell..EM.CD27low.=Q1} => {IL18=Q1}	Low levels of CD8+ EM CD27low T cells are associated with low levels of IL18 cytokine
{CD8..T.Cell..EM.CD27low.=Q1} => {NTF3=Q1}	Low levels of CD8+ EM CD27low T cells are associated with low levels of NTF3 growth factor
{CD8..T.Cell..activated.=Q4} => {NTF3=Q1}	High levels of activated CD8+ T cells are associated with low levels of NTF3 growth factor
{CXCL1=Q1} => {CD4..T.Cell..EMRA.CD57hi.=Q1}	Low levels of CXCL1 cytokine are associated with low levels of CD4+ EMRA CD57hi T cells
{Debris=Q4} => {TNFSF12=Q1}	High levels of cellular debris are associated with low levels of TNFSF12 cytokine
{IL16=Q1} => {CD4..T.Cell..EMRA.CD57hi.=Q1}	Low levels of IL16 cytokine are associated with low levels of CD4+ EMRA CD57hi T cells
{IL18=Q1} => {CD8..T.Cell..EM.CD27low.=Q1}	Low levels of IL18 cytokine are associated with low levels of CD8+ EM CD27low T cells
{NTF3=Q1} => {CD8..T.Cell..EM.CD27low.=Q1}	Low levels of NTF3 growth factor are associated with low levels of CD8+ EM CD27low T cells
{TIMP2=Q1} => {CD4..T.Cell..EMRA.CD57hi.=Q1}	Low levels of TIMP2 protein are associated with low levels of CD4+ EMRA CD57hi T cells
{TNFSF12=Q1} => {Debris=Q4}	Low levels of TNFSF12 cytokine are associated with high levels of cellular debris
{TNFSF8=Q1} => {CD4..T.Cell..EMRA.CD57hi.=Q1}	Low levels of TNFSF8 cytokine are associated with low levels of CD4+ EMRA CD57hi T cells

The identified rules provide insights into the immune mechanisms involved in severe COVID-19 cases and suggest longitudinal trends in immune response dynamics from the point of admission to day 28. Multiple rules indicate that low levels (Q1) of CD4+ T Cell EMRA CD57hi cells are associated with low levels (Q1) of various cytokines such as CXCL1, IL-16, TAFA5, TIMP2, and TNFSF8. The EMRA subset is typically associated with terminal differentiation and senescence. The reduced levels of both these cells and cytokines such as IL-16 and CXCL1 might reflect impaired immune activation or an exhausted T cell phenotype in the severe group. Specific associations highlight low levels of CD8+ T Cell subsets (e.g. EM CD27low and CD161− MAIT cells) with low levels of cytokines like IL-18 and NTF3, suggesting a diminished cytotoxic response in severe cases. Conversely, high levels (Q4) of activated CD8+ T cells are coupled with low levels of NTF3, indicating complex and possibly dysregulated interactions. The strong association between high debris levels and low TNFSF12 levels, as well as other patterns like high CD8+ T cell activation linked to NTF3, align with severe tissue damage and an aberrant cytokine environment. This finding underscores the role of inflammatory milieu and tissue damage in disease severity. The early cytokine environment marked by low levels of IL-16, CXCL1, and IL-18 seems to set the stage for a trajectory toward a compromised immune environment at later time points, which is reflected in the cell subset distributions.

The absence of similarly strong associations in the mild group underscores the distinct immune landscape in severe COVID-19. This difference suggests that severe cases involve more complex and robust immune interactions that are not present or are less pronounced in milder cases. It also implies that early intervention strategies need to be tailored differently based on initial cytokine signatures to effectively manage severe cases.

In contrast, ARM using cell subset data from CyTOF (visit 1 or 6) with cytokine data from Olink (visit 1), which has comparatively lower detectability compared with NULISAseq,^7^ identified only one robust association rule under the same criteria: {CD8+ NKT Cell = Q1} => {CSF1 = Q1} in the combination of Olink visit 1 data and CyTOF visit 6 data. The disparity in the number of identified rules between NULISAseq (17 rules) and Olink (1 rule) assays under the same criteria may highlight the advantage of NULISAseq for its larger panel and/or the increased detectability in detecting meaningful associations. To fairly compare NULISAseq and Olink, the same ARM analysis was performed using common protein targets (n = 65) between the two platforms. Table S4 shows that the number of rules identified using the combination of NULISAseq and CyTOF with a lift threshold of 2.0 was still higher than that identified using Olink and CyTOF with the same lift threshold. Specifically, in trajectory groups 4 and 5 with CyTOF visit 6, 9 of the 11 rules from NULISAseq were included in the 17 rules found in the previous condition, and the 1 rule from Olink was the same as the one identified previously. These findings further underscore NULISAseq’s high detectability in identifying meaningful associations.

## Discussion

The NULISAseq and Olink assays demonstrated reasonable correlation for many shared analytes; however, certain targets reveal that NULISAseq offers a more robust dynamic range and/or enhanced detectability. For researchers aiming for comprehensive analysis, a larger NULISAseq panel may be advantageous for general exploratory purposes. While Olink offers an extensive panel (the Olink Explore option, encompassing over 3,000 targets), both platforms possess unique protein targets. Therefore, the choice of platform should be tailored to the specific requirements and objectives of the research.

Several prominent targets that contribute to the differentiation within the two-dimensional embedding analysis in the star plot have been reported as potential markers of COVID-19 severity and associated complications (e.g. BMP7, CCL1, CEACAM5, IL-1RL1, and SDC1).^28–32^ This observation highlights the utility of NULISAseq with the star plot in detecting potential biomarkers and further enhancing our understanding of their roles in COVID-19 severity.

The elastic net analysis has revealed that a combination of specific targets can effectively predict COVID-19 severity using serum samples at admission. Among these, AREG has been identified previously as a potential marker of COVID-19 severity, corroborating existing literature.^33^ As some studies demonstrated the advantage of large protein target panel to investigate COVID-19 severity,^34^ the use of an extensive panel encompassing 247 targets underscores the power of comprehensive profiling in confirming and expanding upon previously recognized biomarkers, thereby enhancing our understanding of the biological underpinnings of COVID-19 severity.

The longitudinal analysis of COVID-19 patients has revealed significant differences in multiple targets between those with low and high disease severity over time. Notably, higher levels of interferons IFNα-1/13 IFNα-2 were observed in early time points for low-severity trajectory groups 1 to 3 and at later time points for higher-severity trajectory groups 4 and 5, which may align with findings from other studies that highlight their crucial roles in the COVID-19 immune response.^27^ Additionally, IL-5 exhibited higher levels in the severe group across several visits, despite not frequently being reported as a marker for COVID-19 severity. The elevated IL-5 levels may suggest an activation of eosinophils in patients with severe disease, indicating an evolving immune response that could contribute to disease progression. These observations underscore the dynamic nature of the immune response in COVID-19 and emphasize the importance of longitudinal monitoring to uncover evolving biomarkers and therapeutic targets.^35^

In the ARM analysis, combining cytokine data with cell subset data allows for a holistic view of the immune response. Cytokines provide insights into the signaling environment, while cell subsets reveal changes in cellular composition. This dual perspective enhances our understanding of the complex dynamics of disease progression and immune response. ARM identified robust associations between cytokine levels at visit 1 (within 48 h of admission) and cell subset distributions at visit 6 (day 28). These associations indicate that early cytokine signatures can be predictive of later immune cell changes, highlighting the potential for early biomarkers to forecast disease progression. The absence of significant rules linking NULISAseq visit 1 and CyTOF visit 1 data suggests that immediate concomitant measurements may not capture the full complexity of the cytokine-cell subset interactions, emphasizing the importance of a longitudinal perspective. Additionally, understanding the interplay between cytokine levels and cell subset changes can inform the development of tailored therapeutic strategies. For instance, targeting specific cytokines may modulate the immune cell landscape in a beneficial manner, leading to improved patient outcomes.

While this study provides valuable insights into the immune response dynamics in COVID-19 patients, several limitations must be acknowledged. NULISAseq, despite its robust dynamic range and enhanced detectability for certain targets, is limited by the novel technology aspect that may necessitate broader adoption and further validation to ensure reproducibility across different cohorts and laboratories. Although Olink’s panel includes over 3,000 targets, its sensitivity for some analytes may not match that of NULISAseq. The limitation in statistical analysis is that some data may have been missing not at random, for which linear quantile mixed models did not account, so some bias in estimates of temporal trends in medians may have resulted. Additionally, ARM can detect associations between cytokines and cell subsets, but it does not establish causal or mechanistic relationships. This limitation is particularly evident in bidirectional rules, especially those involving different visits (e.g. cytokines from visit 1 and cell subsets from visit 6). Such bidirectional rules could imply that changes in cell subsets at a later time point have an association with earlier cytokine levels, which is not plausible causally and indicates a limitation of ARM in distinguishing temporal causality. Future research should include the validation of NULISAseq results in independent cohorts to ensure the reproducibility and robustness of the findings. Validation will help establish the reliability of potential biomarkers identified in this study and broaden their applicability.

## Supplementary Material

vkaf263_Supplementary_Data

## Data Availability

The data and metadata generated from the IMPACC network were deposited in the ImmPort data repository (www.immport.org) as the primary method of data sharing.
