# Supplementary material for: Evaluating COVID-19 severity prediction and immune dynamics with NULISAseq: Insights from the IMPACC study
Source: J Immunol. 2025 Oct 30;214(12):3310–20. doi: 10.1093/jimmun/vkaf263 (PMC12726064; doi:10.1093/jimmun/vkaf263)
Supplement: vkaf263_Supplementary_Data [file vkaf263_supplementary_data.pdf]

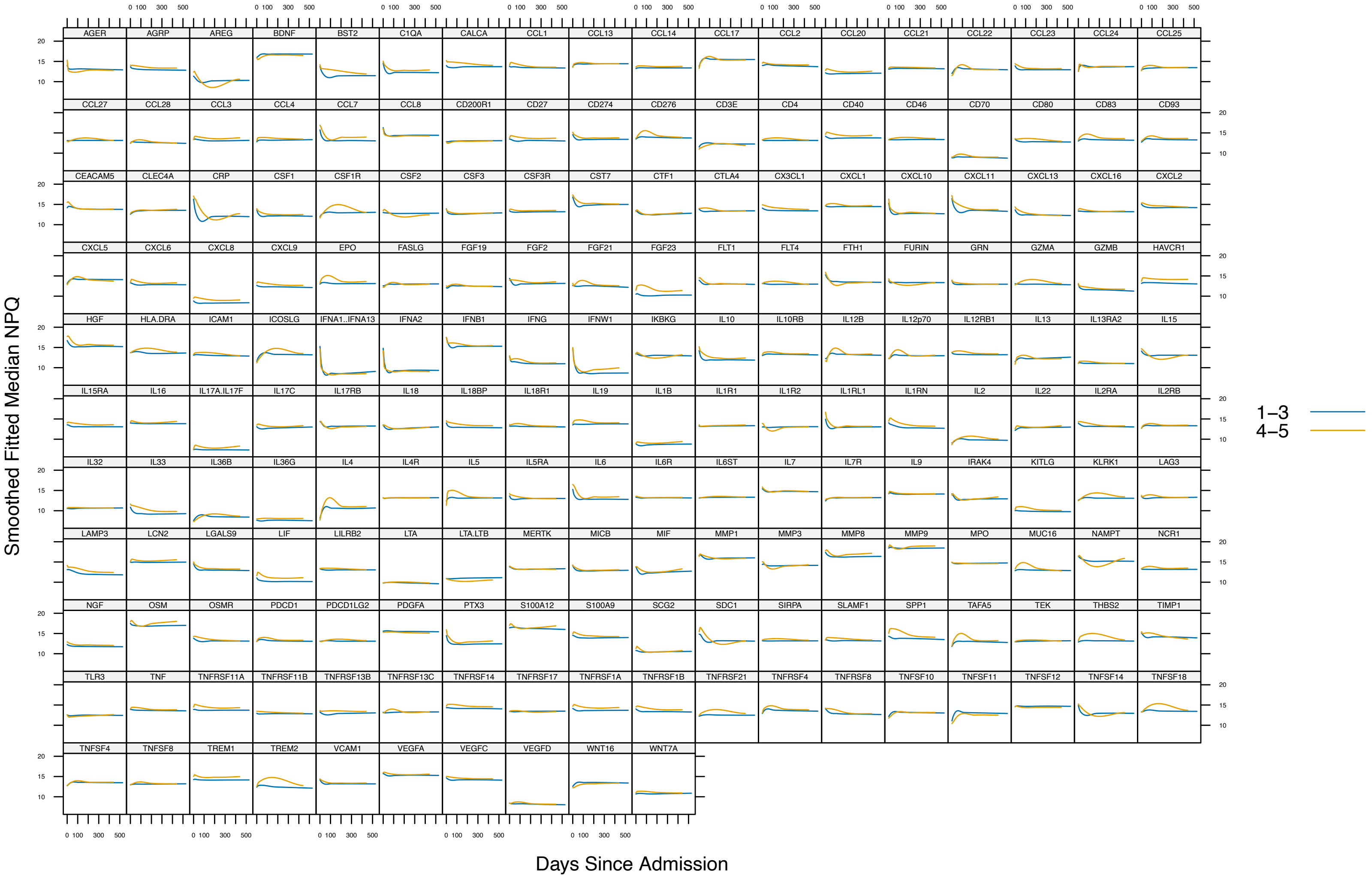

Supplemental Figure 1. A total of 190 proteins from NULISAseq exhibiting statistically significant differences in longitudinal trends between severity groups. Due to high statistical power, even subtle differences could be detected as significant. The smooth fitted median NPQ represents the loess-smoothed LQMM fitted values of median NULISA expression, with smoothing over age and enrollment site.

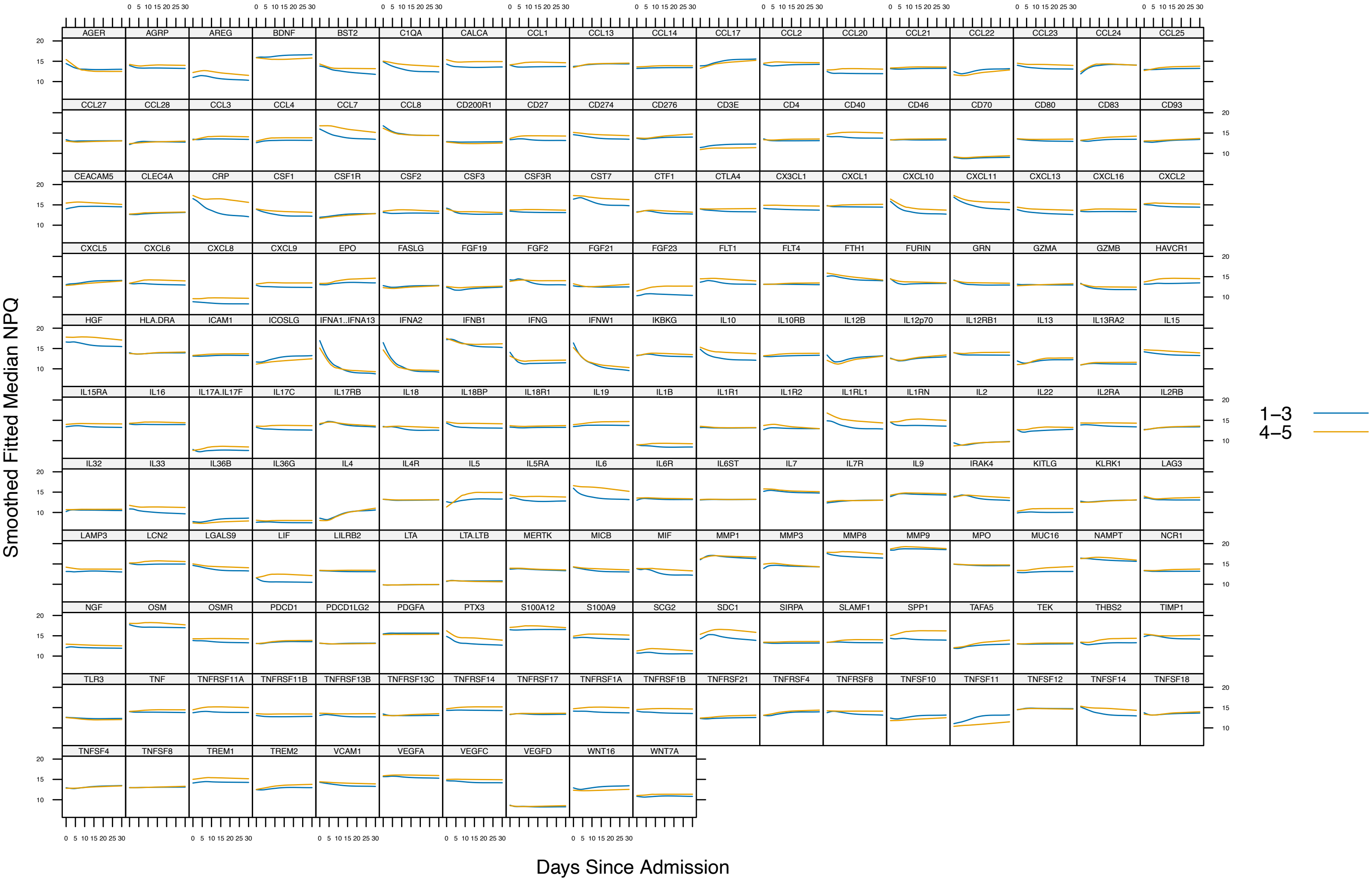

Supplemental Figure 2. Longitudinal analysis with linear quantile mixed models for modeling trends in the median within the first 30 days using NULISAseq data. The fit shown for the first 30 days is an excerpt of the fit obtained from modeling trends across all days. Due to high statistical power, even subtle differences could be detected as significant. The smooth fitted median NPQ represents the loess-smoothed LQMM fitted values of median NULISA expression, with smoothing over age and enrollment site.

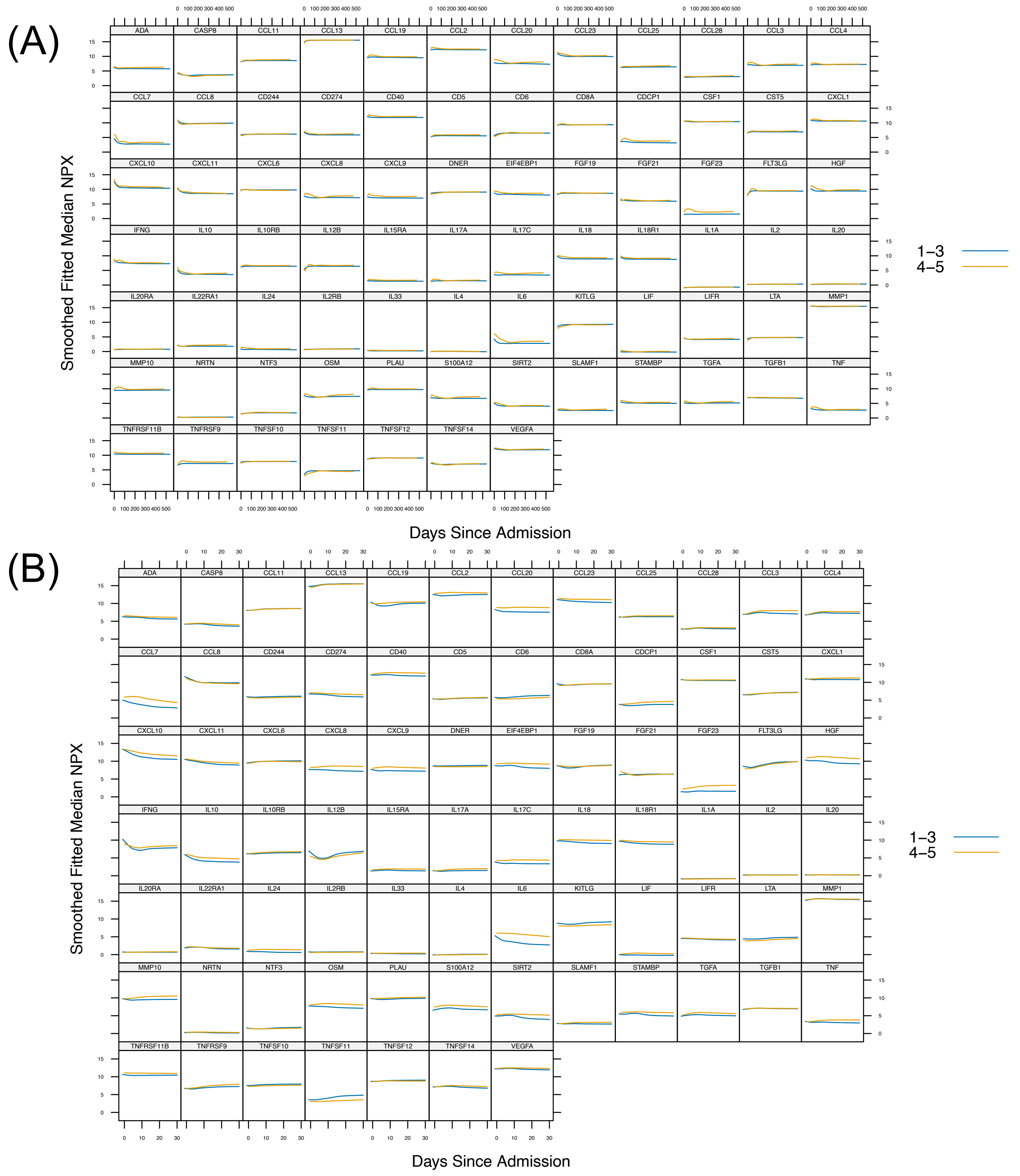

Supplemental Figure 3. Longitudinal analysis using Olink data. Statistically significant proteins with likelihood ratio test statistics in the upper quartile are shown. (A) A total of 79 proteins from Olink exhibiting statistically significant differences in longitudinal trends between severity groups. (B) Longitudinal analysis with linear quantile mixed models for modeling trends in the median within the first 30 days. The fit shown for the first 30 days is an excerpt of the fit obtained from modeling trends across all days. Due to high statistical power, even subtle differences could be detected as significant. The smooth fitted median NPX represents the loess-smoothed LQMM fitted values of median Olink expression, with smoothing over age and enrollment site.

Supplemental Table I. The number of observations in each combination of visits from NULISAseq and CyTOF data

NULIS	Visit 1									
CyTOF	Visit	Visit	Visit	Visit	Visit	Visit	Visit	Visit	Visit	Visit 10
#	328	207	136	84	44	66	20	20	20	11

Supplemental Table II. The number of rules found in Case A (NULISAseq: Visit 1, CyTOF: Visit 1) and Case B (NULISAseq: Visit 1, CyTOF: Visit 6)

	Case A		Case B	
Trajectory group	1-3	4-5	1-3	4-5
Lift > 1.0	103	309	173	187
Lift > 1.5	1	1	15	36
Lift > 2.0	0	0	0	17

Supplemental Table III. The number of observations in each combination of visits from Olink and CyTOF data

Olink	Visit 1									
CyTOF	Visit	Visit	Visit	Visit	Visit	Visit	Visit	Visit	Visit	Visit 10
#	734	356	219	158	57	147	83	129	145	126

Supplemental Table IV. The number of rules found by ARM with common protein targets between NULISAseq and Olink

		CyTOF (Visit 1)		CyTOF (Visit 6)	
	Trajectory	1-3	4-5	1-3	4-5
NULISAseq (Visit 1)	Lift > 1.0	35	85	56	72
	Lift > 1.5	0	1	12	21
	Lift > 2.0	0	0	3	11
Olink (Visit 1)	Lift > 1.0	28	108	32	78
	Lift > 1.5	0	1	0	8
	Lift > 2.0	0	0	0	1
